# Phenotypes of macrophages present in the intestine are impacted by stage of disease in cattle naturally infected with *Mycobacterium avium* subsp. *paratuberculosis*

**DOI:** 10.1371/journal.pone.0217649

**Published:** 2019-05-23

**Authors:** Caitlin J. Jenvey, Adrienne L. Shircliff, John P. Bannantine, Judith R. Stabel

**Affiliations:** USDA-Agricultural Research Service (ARS), National Animal Disease Center, Ames, IA, United States of America; Cornell University, UNITED STATES

## Abstract

Macrophages play an important role in the host immune response to *Mycobacterium avium* subsp. *paratuberculosis* (MAP) infection, however, MAP is able to disrupt normal macrophage functions to avoid destruction. It is unclear whether the phenotypes of macrophages present in the target tissue play a role in the inability to clear MAP infection. The aim of this study was to identify macrophage phenotypes (host defense or resolution and repair) present within the bovine ileum of naturally infected cattle, as well as to ascertain abundance of each macrophage phenotype present during different stages of MAP infection. Immunofluorescent (IF) labeling was performed on frozen bovine mid-ileal tissue sections collected from 28 Holstein dairy cows. Comprehensive IF staining for cytokines, such as IFN-γ, IL-1Ra, IL-1β, IL-10, TGF-β, TNF-α, and uNOS, along with markers such as CD163, CD206, and TLR4, served to define the macrophage phenotypes. Overall, cows in the clinical stage of disease demonstrated significantly higher numbers of resolution and repair macrophages and lower numbers of host defense macrophages in the ileal tissue. Interestingly, subclinically affected cows with asymptomatic disease had a nearly equal ratio of host defense and resolution and repair macrophage phenotypes, whereas macrophage phenotype was skewed to a host defense macrophage in the tissues of the control noninfected cows. The preponderance of M2-like resolution and repair phenotype for macrophages in the tissues of cows with clinical disease would explain why the host fails to control and/or clear the infection, leading to a higher MAP burden. The results of the current study offer insight into the disparate macrophage phenotypes present in the bovine ileum during different stages of infection.

## Introduction

Macrophages play an important role in the host immune response to infection with bacterial pathogens. In particular, *Mycobacterium avium* subsp. *paratuberculosis* (MAP), an intracellular pathogen that causes enteritis in ruminants, is engulfed by intestinal macrophages as part of the initial response to infection. The ability of macrophages to destroy the invading pathogen is a critical component of host defense and determines whether the host will clear the infection or eventually succumb to clinical disease. Macrophages may be categorized as either classically activated (M1) or alternatively activated (M2). More definitively, the M1 macrophage encompasses a pro-inflammatory host defense phenotype, whereas M2 macrophages embody both wound-healing (tissue repair) and regulatory phenotypic functions [1] Polarization of macrophages between phenotypes is a dynamic process, with the dominance of a phenotype dependent upon the presence and persistence of stimuli [1–3] Indeed, a plasticity of macrophage phenotypes exists during the infectious process with heterogeneous populations of macrophages observed in mycobacterial infections [4,5]. Furthermore, it is suggested that mycobacteria may be involved in selective recruitment of macrophages [4], as well as inhibition of macrophage inflammatory response [6] that enable them to survive in the host.

Macrophages use pattern recognition receptors to recognize MAP during the infectious process and begin phagocytosis [1,7]. Phagocytosis by intestinal macrophages does not initially induce cytokine release, thus, resident intestinal macrophages perform their host defense activities without inducing an inflammatory response [4]. Although these macrophages readily phagocytize the pathogen, phagocytosis often does not result in killing of the pathogen. The functions and/or responsiveness of the macrophage can be disrupted by MAP through a variety of methods, including preventing macrophage activation [8–11], blocking phagosome maturation [12–14], preventing macrophage acidification [12,15], and delaying macrophage apoptosis [13,16]. Although host defense macrophages are vital first responders to bacterial infection, a prolonged inflammatory response can be harmful to the host [2]. In Johne’s disease, initial infection with MAP results in a subclinical infection where little intestinal inflammation is observed, whereas the more advanced stages of infection are characterized by extensive levels of inflammation with an influx of macrophages to the target tissue [17]. The progression from subclinical to clinical stages of disease may be a result of the polarization of macrophage phenotype from a host defense phenotype towards a regulatory phenotype to help control the inflammation. Macrophage phenotypes can be characterized using secretion of cytokines and expression of cell surface markers, such as macrophage receptors. Host defense macrophages (M1) are characterized by expression of pro-inflammatory cytokines such as IFN-γ, IL-1β, IL-12, IL-23, and TNF-α, whereas resolution/regulatory and repair macrophages are characterized by expression of the surface markers, CD163, CD206, and cytokines IL-1Ra, IL-10, and TGF-β [1]. The aim of the present study was to identify macrophage phenotypes present within the bovine ileum of naturally infected cattle, as well as to characterize relationships between the macrophage phenotypes present during subclinical and clinical stages of MAP infection.

## Materials and methods

### Animals

Samples of mid-ileal tissue were collected at necropsy from a total of 20 Holstein dairy cows naturally infected with MAP, and 8 uninfected control cows. Cows were maintained as part of a herd housed at the National Animal Disease Center (NADC, Ames, IA), and were placed in three groups consisting of 8 non-infected healthy cows, 10 cows that were subclinically affected, and 10 cows with the clinical form of the disease with an average age of 7 (range 3 to 10), 7 (range 3 to 13), and 5.7 (range 4 to 9) years of age, respectively. Control uninfected cows used in the present study were obtained from accredited JD-free herds and/or raised onsite from test-negative dams. Infected animals were either purchased from herds known to be positive for Johne’s disease or were replacement animals born onsite to infected dams. Infected animals were naturally infected with MAP and were classified into the subclinical and clinical categories based upon fecal shedding, determined by fecal culture and PCR, and clinical signs [18]. Infected and control cows were housed separately at the NADC and tested at least biannually, with endpoint testing just prior to necropsy [19]. Cows were housed in the dairy cattle barn onsite with access to freestall and pasture areas for resting. Cows were fed a total mixed ration consisting of chopped grass hay, soybean meal, cracked corn, and corn silage on an ad libitum basis and free access to automated waterers and mineral blocks. Animals were milked and fed twice per day and monitored for health events at that time. In the event of unexpected illness or injury animals were treated appropriately with analgesics by the clinical veterinarian. Prior to necropsy, infection was monitored bacteriologically for fecal shedding of MAP using fecal culture and PCR as previously described [18], as well as serologic tests, such as Herdchek ELISA for serum antibodies (IDEXX, Westbrook, ME) and a modified MAP-specific IFN-γ assay measured in the plasma (Bovigam, Thermo Fisher Scientific, Carlsbad, CA) [20]. Animals categorized as clinical demonstrated serum ELISA antibody titers averaging 2.45 S/P ratio and fecal shedding average 2,369 CFU of MAP/g feces. Cows in the subclinical group were ELISA-negative and averaged less than 17 CFU of MAP/g feces. Infected animals in both the subclinical and clinical stages of infection had equivalent positive antigen-specific IFN-γ results (Abs_450nm_MPS-Abs_450nm_NS = 0.20). There was no recorded evidence of any other health issues for cows used in this study that would impact the results. All uses of animals in this study were approved by the institutional guidelines and animal care and use committee (IACUC), protocol#ARS-2018-722 (NADC).

### Tissue collection and processing

Animals were euthanized via intravenous administration of sodium pentobarbital (60 mg/kg body weight) to overdose and then exsanguinated according to IACUC guidelines. At necropsy, the entire section of ileum extending from the ileo-cecal valve through the distal flange was excised and then cut equally into proximal, mid- and distal sections. Tissues were rinsed with 0.15 M PBS and cut into multiple cross-sections for culture of MAP and PCR to assess bacterial burden. Cross-sections immediately adjacent were processed for histopathology and immunofluorescent (IF) labeling. A dry ice bath was prepared by combining 95% ethanol with dry ice and mixed until a slurry consistency was achieved. Isopentane (Sigma-Aldrich, St. Louis, MO) was added to a tin cup and the cup was placed into the dry ice bath. The mid-ileal intestinal samples were washed with PBS, pH 7.4, and a cross-section was positioned luminal side down on a section of liver covered with Tissue-Tek optimum cutting temperature (OCT) compound (Sakura Finetek, Torrance, CA) in order to protect the villi during the freezing process and to ascertain tissue orientation post-freezing. The intestine-liver sample was wrapped in foil and placed in the isopentane for at least 5 minutes. The snap-frozen sample was transferred to dry ice for transport to storage at -80°C, where it remained until tissue sectioning could be performed. Tissues were also fixed, stained and examined for acid-fast (AF) bacteria and granulomatous lesions. Briefly, no granulomas were present in the ileal tissues from control cows and present in only 1 of 10 subclinical cows, with rare small foci of macrophages in the villus tips. In contrast, granulomas were present within the lamina propria and submucosa in tissue from 7 of the 10 clinically affected cows, with macrophages located commonly around submucosal lymphatic vessels. The presence of AF bacteria was noted only clinically affected cows with scores ranging from 0 (none) to 5 (multibacillary). The 10 cows in the clinical group were randomized across the AF scoring with 3, 0, 2, 3, 1, and 1 animal per each scoring rank from 0 to 5. Tissue culture and PCR results delineating tissue burden of MAP was greater in clinically affected cows compared to subclinical cows and has been previously described [21].

#### Tissue sections

The mid-ileal intestinal samples were removed from -80°C and placed in a cryostat at -20°C for at least 30 minutes prior to sectioning. Tissue samples were embedded in OCT, cut in 6 μm sections and adhered to ColorFrost Plus microscope slides (Thermo Fisher Scientific, Carlsbad, CA). Tissue sections were allowed to air-dry overnight at room temperature before fixing for 5 minutes at -20°C. Tissue sections were stored at -80°C until histochemistry and IF staining could be performed.

### Immunofluorescence protocol

Tissue sections were removed from -80°C and allowed to equilibrate to room temperature for 10–20 minutes. A liquid blocker ‘Pap’ pen was used to draw a hydrophobic barrier around the tissue and allowed to dry. Following tissue rehydration, 3,3-diaminobenzidine (DAB, Vector Laboratories, Burlingame, CA) was added for 10 minutes to quench eosinophil autofluorescence and washed 3 times for 5 minutes each. A protein, serum-free blocking solution (Agilent Dako, Santa Clara, CA) was added for 30 minutes to reduce non-specific labeling. The slides were not washed in-between serum blocking and endogenous biotin blocking/primary antibody incubation. For protocols that included biotinylated primary antibodies, slides were blocked for endogenous biotin prior to the incubation of the first biotinylated primary antibody, and in-between biotinylated primary antibodies. Streptavidin and biotin (ThermoFisher Scientific, Carlsbad, CA) were diluted to concentrations of 0.1mg/ml and 0.05mg/ml, respectively. Streptavidin was incubated first for 15 minutes, followed by a washing step. Slides were washed alternately with 0.05M Tris buffer, followed by 0.05M Tris buffer with 0.02% Tween-20 and 0.9g sodium chloride, 3 times for 3 minutes each. Following washing, biotin was incubated for 15 minutes, followed by a second washing step. The primary and secondary antibodies, their dilutions and incubation times are presented in Tables 1 and 2, respectively. Primary antibodies were biotinylated using an EZ-Link Sulfo-NHS-LC-Biotin and procedure outlined by ThermoFisher Scientific (Carlsbad, CA). Briefly, 2 mg of biotin was diluted in ultrapure water at 1:25. An appropriate volume of 1:25 biotin solution was added to 100 μL of primary antibody and allowed to incubate at room temperature for 60 minutes. The biotinylated primary antibodies were added to a 0.5 mL desalting column and centrifuged at 1500 x g for 2 minutes to purify the biotinylated antibody. The slides were washed after primary and secondary antibody incubation using the method described above. Incubation with a 1:6,000 dilution of 4’,6- diamidino-2-phenylindole, dihydrochloride (DAPI) for 10 minutes was used to counterstain the nuclei. The slides were mounted in ProLong Gold Antifade Mountant (Thermo Fisher Scientific, Carlsbad, CA) and Richard-Allen Scientific ‘Slip-Rite’ Cover Glass #1.5 (Thermo Fisher Scientific, Carlsbad, CA). The mounting medium was allowed to cure for at least 30 minutes at room temperature before imaging.

**Table 1:** Primary antibodies used in study.

Antibody	Clonality	Host	Reactivity	Isotype	Dilution	Manufacturer
AM-3K	M	Mouse	C, H, Do, Ca, Ho, P, R	IgG_1_	1:200	Abnova, Taiwan
CD163	M	Mouse	C, G, S	IgG	1:200	WSU, WA
CD206^a^	M	Mouse	H, P, S	IgG_1_	1:50	Novus Biologicals, CO
IFN-γ^a^	M	Mouse	C, D, G	IgG1	1:50	BioRad, CA
IL-10^a^	M	Mouse	H	IgG2b	1:20	Santa Cruz Biotech, TX
IL-12p40 ^a^	M	Mouse	C, H, S, AB	IgG2a	1:50	BioRad, CA
IL-1Ra^a^	P	Rabbit	C	IgG	1:30	Cloud-Clone Corp., TX
IL-1β^a^	M	Mouse	C, S, G	IgG1	1:50	LifeSpan Biosci, WA
IL-23^a^	M	Mouse	H, Pr	IgG_1κ_	1:50	Novus Biologicals, CO
TGF-β^a^	M	Mouse	C, Ch, M, H	IgG1	1:50	Novus Biologicals, CO
TLR4	P	Rabbit	C, H, M, Ra	IgG	1:200	Bioss Antibodies, MA
TNF-α^a^	P	Rabbit	C, Ch, Dk, Ho, Hm, P, R	IgG	1:50	Abbiotec, CA
uNOS^a^	M	Mouse	C, H, M, Ra	IgM	1:100	Novus Biologicals, CO

^a^Antibodies biotinylated in-house. Abbreviations: AB, African Buffalo, C, Cattle; Ca, Cat; Ch, Chicken; D, Deer; Dk, Donkey; Do, Dog; H, Human; Hm, Hamster; Ho, Horse; G, Goat; M, Mouse; P, Pig; Pr, Primate; R, Rabbit; Ra, Rat; S, Sheep.

**Table 2:** Secondary antibodies used in study^a^.

Antibody	Reactivity	Host	Isotype	Dilution
Alexa Fluor 555	Mouse	Goat	IgG_1_	1:1000
Alexa Fluor 647	Rabbit	Goat	IgG (H+L)	1:1000
Alexa Fluor 647	Mouse	Goat	IgG (H+L)	1:500
Alexa Fluor 488 Conjugation Kit	N/A	N/A	N/A	N/A
DyLight 488	NeutrAvidin	N/A	N/A	1:200
DyLight 594	NeutrAvidin	N/A	N/A	1:200

^a^ Thermo Fisher Scientific, CA.

### Confocal imaging

The tissue sections were examined with an A1 Resonance Plus inverted microscope (Nikon, Melville, NY) equipped with a four-laser Gallium-Arsenide-Phosphide/normal Photomultiplier Tube detector unit (DU4) (GaAsP: 488 and 561; PMT: 405 and 640), Galvano resonant scanner and NIS Elements Advanced Research software (version 4.50.00). Images were acquired by sequential scanning to avoid fluorescence cross-over using a 405/488/561/640 dichroic mirror. All slides were imaged using the following bandpass filters: 405 solid-state diode laser and 450/50nm bandpass filter, 488nm solid-state diode laser and 525/50 bandpass filter, 561nm solid-state diode laser and 600/50 bandpass filter, and 640 solid-state diode laser and 685/70nm bandpass filter. Images were captured using a 60x Plan Apo lambda objective (1024 x 1024 pixels), numerical aperture 0.75, pinhole 1.2 AU, and exposure 6.2 seconds per pixel dwell. Detector sensitivity (gain) and laser power settings were kept the same for all collected images to allow comparisons between markers and cows. A total of 10 images were collected per cow to perform statistical analysis.

#### Determination of macrophage number by surface area labeling

Upon collection of each image, spectral profiles for each primary antibody were determined based upon slides containing only a single primary and secondary antibody. Spectral profiles were used to subtract true IF labeling from background. Thresholding was then performed to create a binary layer for each laser channel on which quantitative analysis of IF labeling could be performed. The lower and upper intensity limits were thresholded to reduce the contribution of non-specific immunofluorescent (IF) labeling to binary layer calculations. Additionally, binary layer contours were smoothed and cleaned to remove small objects and reconstruct morphology. Macrophage phenotypes were determined by measurement of surface/cytokine markers overlapping with macrophage labeling (co-localization) within 10 images per cow. The total surface area (μm^2^) for the AM-K3 positive macrophages was imaged first, followed by co-localization of fluorescent markers within the AM-K3 positive macrophages. A macro was developed (Nikon Instruments Support Team, Minneapolis, MN) to automatically measure the surface area of each surface/cytokine marker overlapping with AM-K3 positive macrophages in all collected images, based upon the pre-determined spectral profiles and thresholding settings. All data were collated in Microsoft Excel prior to statistical analysis.

### Statistical analysis

Frequency histograms and Q-Q plots determined that the data was non-normally distributed with a positive skew. A Log10 transformation was performed to achieve normally distributed data. Box plots were used to identify outliers, which were removed from the dataset if greater than 2 standard deviations above the mean. Mean ± 95% confidence intervals for each antibody or combinations of antibodies was calculated for each cow, and for each group (control, subclinical, clinical). Mean macrophage was divided by mean T-cell to calculate the ratio of macrophages to T-cells for each cow.

An analysis of variance (ANOVA) with a post-hoc Tukey’s test was performed to determine significant differences (Pr>F = 0.05) within and between groups. A pair-wise Pearson correlation coefficient (Pr>0.05) was calculated to identify significant relationships between continuous variables. Statistical analysis was performed using JMP version 12.

## Results

### Host defense macrophages present in tissue

**Fig 1:**
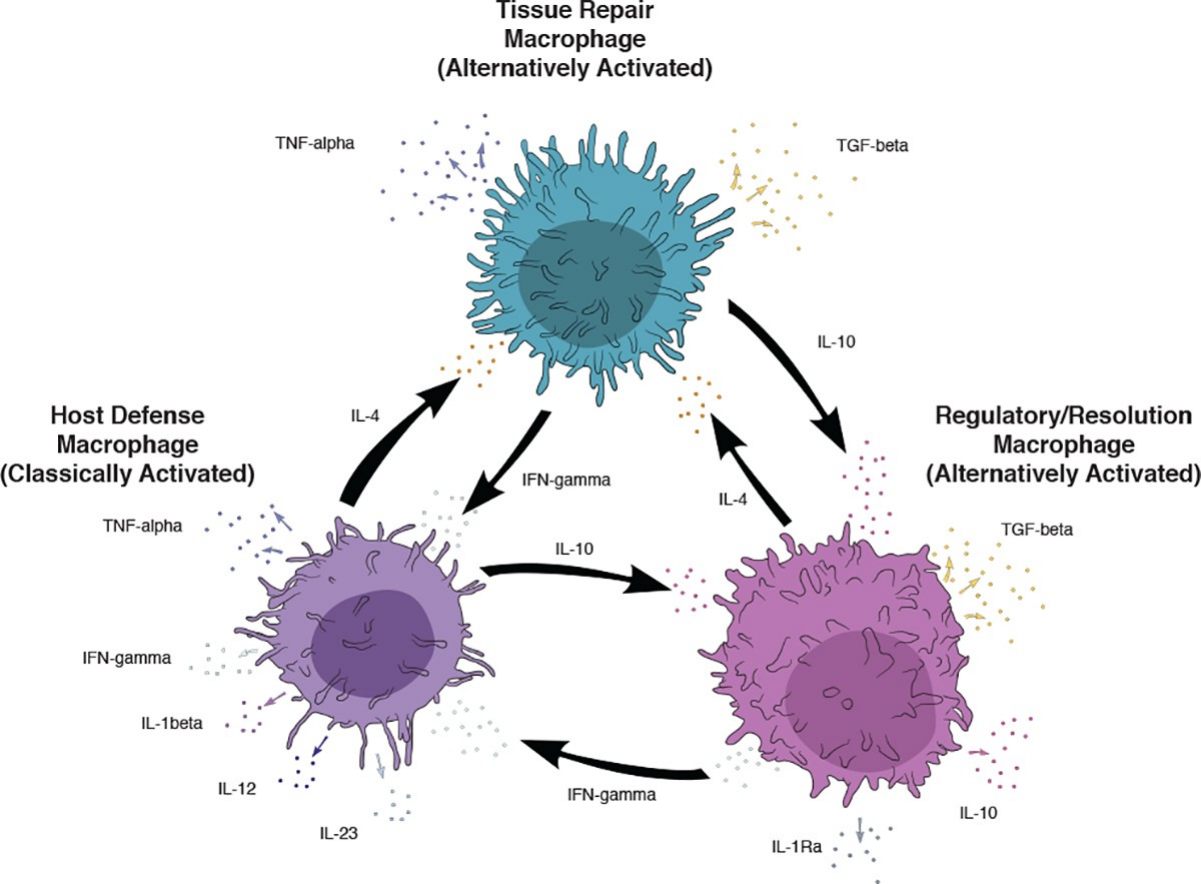
Schematic outlining relationships and properties of polarized macrophages.

**Fig 2:**
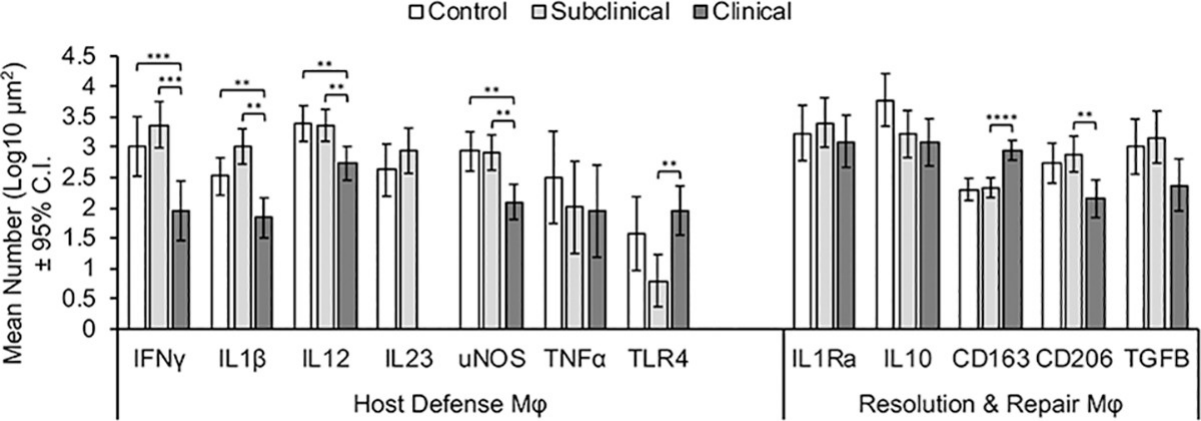
Mean number (Log μm^2^) of macrophages (AM-3K) classified as host defense (IFN-γ, IL-1β, IL-12, IL-23, uNOS, TNF-α, and TLR4) or regulation of inflammation (IL-1Ra, IL-10m CD163, CD206, and TGF-β), present in frozen bovine mid-ileal tissue sections collected from cows naturally infected with *Mycobacterium avium* subsp. *paratuberculosis* (subclinical and clinical), compared to uninfected control cows. Asterisks denote statistical significance: * p<0.05; ** p<0.01; *** p<0.001; **** p<0.0001.

Host defense macrophages (M1) are characterized by expression and/or secretion of the following surface markers and cytokines: IFN-γ, IL-1β, IL-12, IL-23, uNOS, TNF-α, and TLR4 (Fig 1). Cows with clinical disease demonstrated significantly lower mean numbers of macrophages that colocalized with IFN-γ (p = 0.001), IL-1β (p = 0.0002), IL-12 (p = 0.005), and uNOS (p = 0.0007) when compared to either control or subclinical cows (Fig 2). In contrast, clinical cows demonstrated significantly higher mean numbers of macrophages that colocalized with TLR4 (p = 0.005) when compared to subclinical cows (Fig 2). Further, there was no demonstrable IL-23 present in the tissue of clinically affected cows when compared to either control or subclinical cows, and there were no differences in the number of macrophages associated with TNF-α expression. There were no significant differences between control and subclinical cows as results for co-localization of macrophages and the aforementioned markers were closely aligned for these two treatment groups (Fig 2).

### Resolution and repair macrophages present in tissue

**Fig 3:**
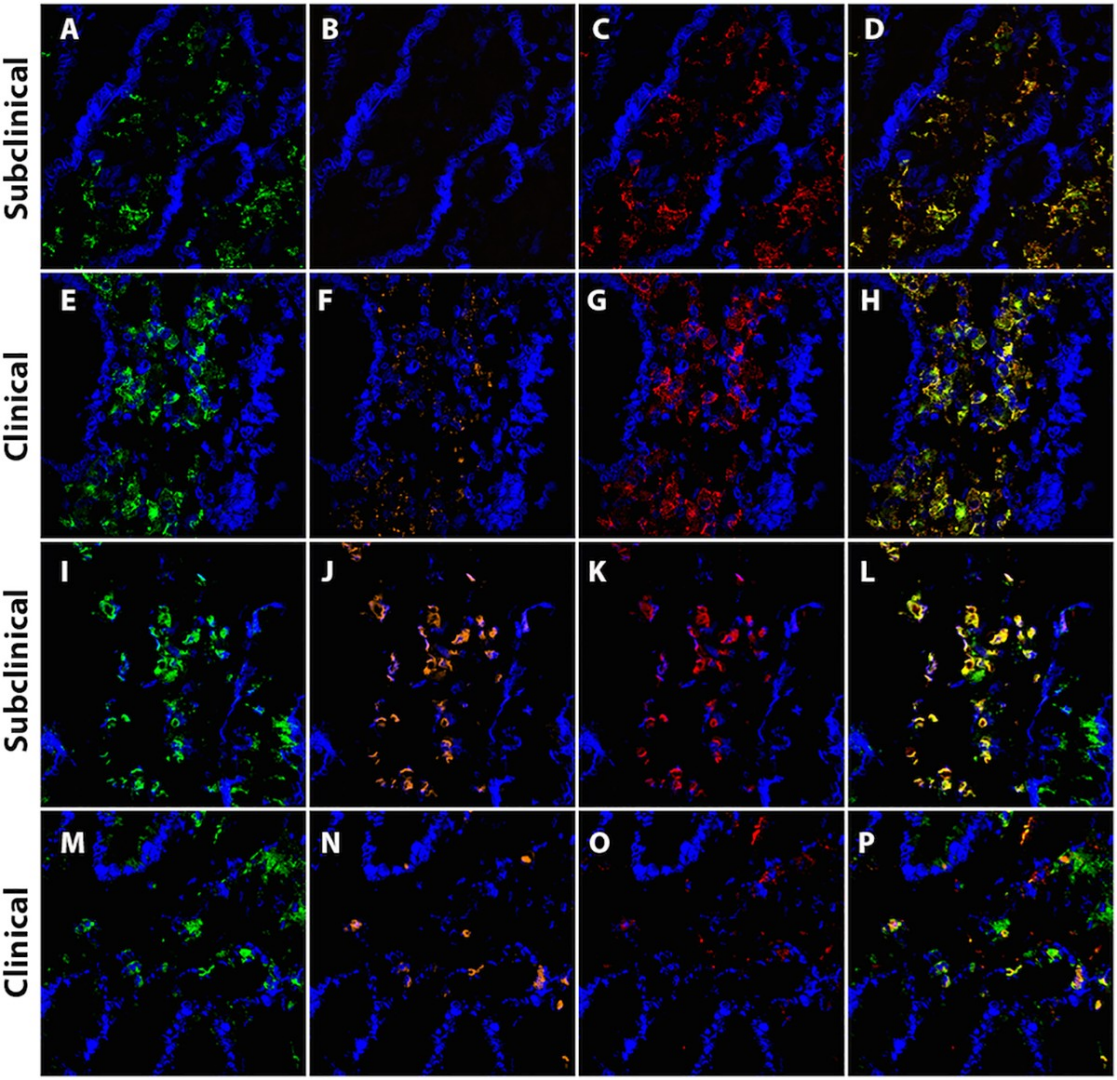
Representative IF labeling in frozen bovine mid-ileal tissue, comparing cows subclinically and clinically infected with *Mycobacterium avium* subsp. *paratuberculosis* (MAP). Nuclear staining was performed using 4’,6- diamidino-2-phenylindole, dihydrochloride (DAPI, blue). Each merged image was selected as an example of representative IF labeling from a single cow. (A and E) CD163; (B and F) TLR4; (C and G) AM-3K; (D and H) merged image of CD163, TLR4 and AM-3K; (I and M) CD206; (J and N) uNOS; (K and O) AM-3K; (L and P) merged image of CD206, uNOS and AM-3K.

Resolution/regulatory and repair macrophages are characterized by expression/secretion of the surface markers and cytokines: CD163, CD206, IL-1Ra, IL-10, and TGF-β (Fig 1). In the present study, clinically affected cows demonstrated significantly lower mean numbers of macrophages with CD206 (p = 0.007) expression, and significantly higher expression of CD163 (p<0.0001) on macrophages compared to subclinical cows and control noninfected cows (Fig 2). Additionally, a strong though nonsignificant trend existed for lower TGF-β expression on macrophages for clinically affected cows. The relative colocalized staining patterns for regulatory macrophages were very similar for control noninfected cows and subclinically affected cows, as previously noted for host defense macrophages. Representative IF labeling for AM-3K only (macrophage), as well as colocalized staining with AM-3K and CD163, TLR4, uNOS, and CD206 in subclinical and clinical cows is presented in Fig 3.

**Fig 4:**
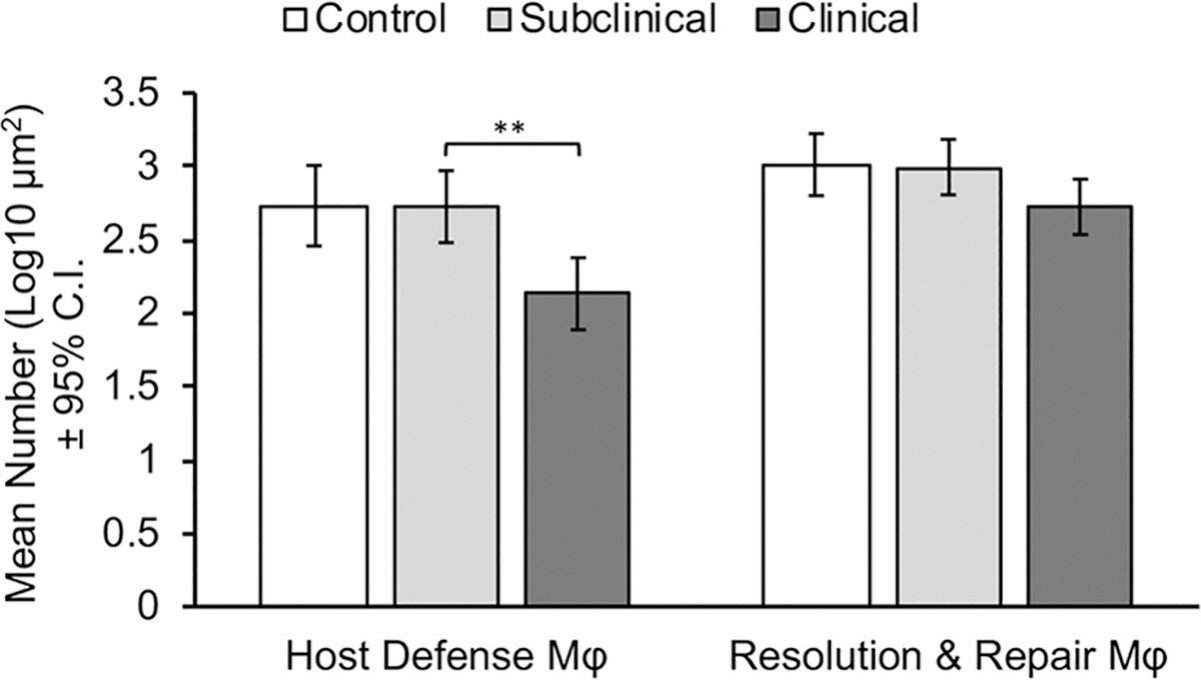
Mean number (Log μm^2^) of total macrophages (AM-3K) classified as either host defense or regulation of inflammation macrophages. IF staining was performed on frozen bovine mid-ileal tissue sections collected from cows naturally infected with *Mycobacterium avium* subsp. *paratuberculosis* (subclinical and clinical), compared to uninfected control cows. Asterisks denote statistical significance: ** p<0.01.

Macrophages with individual markers as defined above were grouped into total host defense and total resolution and repair macrophage phenotypes, and differences between control, subclinical and clinical cows were assessed. Overall, clinical cows demonstrated significantly lower mean numbers of macrophages with host defense phenotypes when compared to control and subclinical cows (p = 0.0032). Although clinical cows also demonstrated reduced numbers of macrophages with a regulatory phenotype, this reduction was not significantly different from control and subclinical cows (Fig 4).

### Macrophage-cytokine correlation

**Fig 5:**
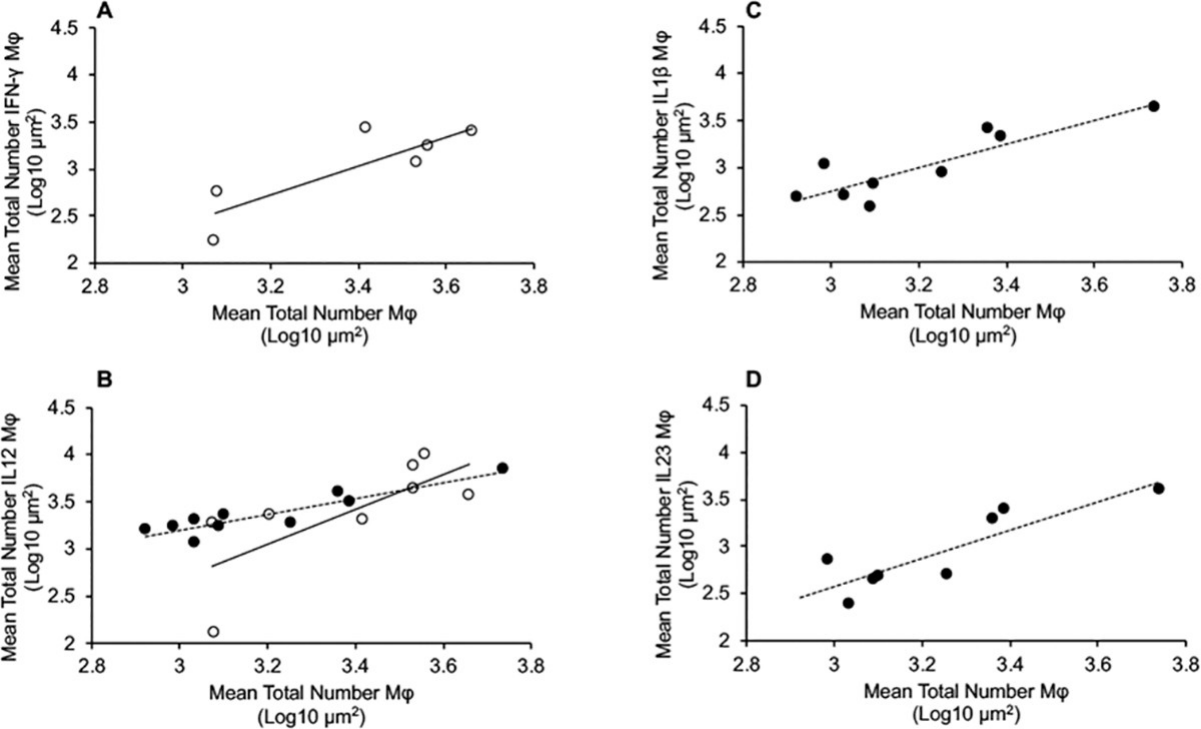
Pairwise Pearson correlations for total number of macrophages (Mφ) with host defense Mφ phenotypes. (**A**) correlation between total Mφ and number of IFN-γ+ Mφ for control cows; (**B**) correlation between total Mφ and number of IL-12+ Mφ for control cows (○ and solid line) and subclinical cows (● and dotted line); (**C**) correlation between total Mφ and number of IL-1β+ Mφ for subclinical cows; and (**D**) correlation between total Mφ and number of IL-23+ Mφ for subclinical cows).

Pairwise Pearson correlations were performed to assess the relationships between IF staining for the macrophage and specific cytokines. Correlations are first presented as total macrophages that grouped together within the host response phenotype versus macrophages that are positive for cytokines that are commonly associated within that phenotype, such as IFN-γ, IL-1β, IL-12, IL-23, and uNOS by treatment group. Mean numbers of total macrophages demonstrated significant and positive correlations with mean numbers of IFN-γ+ (r = 0.842, p = 0.036) and mean numbers of IL-12+ (r = 0.738, p = 0.037) macrophages for control cows (Fig 5A and 5B). Subclinical cows also demonstrated positive correlations between total macrophages and IL-12+ macrophages (r = 0.872, p = 0.001) (Fig 5B), in addition to significant and positive correlation between IL-1β+ macrophages (r = 0.872, p = 0.002) (Fig 5C), and IL-23+ macrophages (r = 0.873, p = 0.005) (Fig 5D). In addition, correlations were made between macrophages that grouped into the host defense phenotype by cytokine (i.e, those that stained positive for IFN-γ, IL-1β, IL-12, IL-23, and uNOS) within each disease state. Subclinical cows demonstrated the most significant number of host defense phenotype correlations (7 additional correlations), that included correlations between macrophages colocalized with IL-12, IL-23 and uNOS and IL-10+ macrophages (Table 3). Control cows demonstrated an additional 5 correlations, with correlations between macrophages positive for IFN-γ and IL-1β demonstrating significant positive correlations with IL-23, uNOS and IL-12 (Table 3). Interestingly, clinically affected cows did not demonstrate any significant correlations within host defense phenotype macrophages (Table 3).

**Table 3:** Pairwise Pearson correlations between cytokines colocalized within host defense macrophages.

Dependent variable	Independent variable		Pearson correlation (r)	P-value	
Control cows					
Mφ ALL	Mφ IFN-γ		0.842	0.036	
Mφ ALL	Mφ IL-12		0.738	0.037	
Mφ IFN-γ	Mφ IL-23		0.966	0.007	
Mφ IFN-γ	Mφ IL-1β		0.844	0.019	
Mφ IFN-γ	Mφ uNOS		0.933	0.007	
Mφ IL-1β	Mφ uNOS		0.872	0.005	
Mφ IL-1β	Mφ IL-12		0.813	0.049	
Subclinical cows					
Mφ ALL	Mφ IL-12		0.908	0.000	
Mφ ALL	Mφ IL1-β		0.872	0.002	
Mφ ALL	Mφ IL-23		0.873	0.005	
Mφ IL-1β	Mφ IL-23		0.953		0.000
Mφ IL-1β	Mφ IL-12		0.904		0.001
Mφ IL-23	Mφ IL-12		0.880		0.004
Mφ uNOS	Mφ IL-12		0.663		0.037

Abbreviations: Mφ, macrophage.

**Fig 6:**
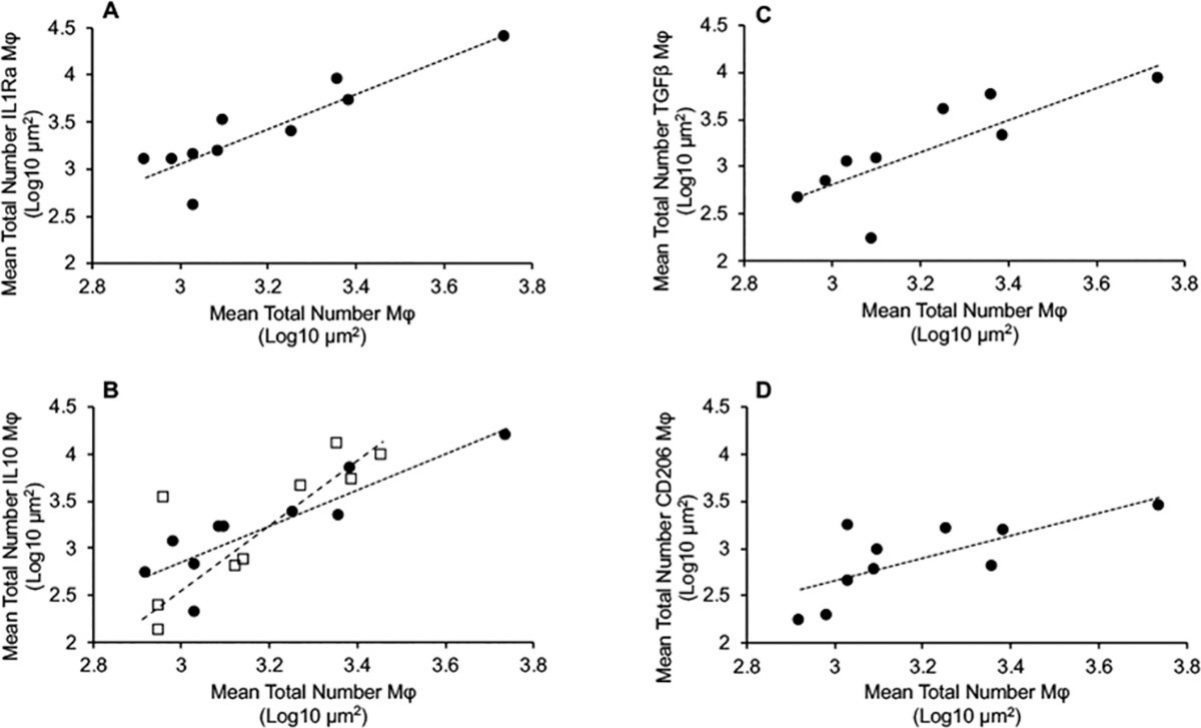
Pairwise Pearson correlations for total number of macrophages (Mφ) with resolution and repair Mφ phenotypes. (**A**) correlation between total Mφ and number of IL-1Ra+ Mφ for subclinical cows; (**B**) correlation between total Mφ and number of IL-10+ Mφ for subclinical cows (● and dotted line) and clinical cows (□ and dotted line); (**C**) correlation between total Mφ and number of TGF-β+ Mφ for subclinical cows; and (**D**) correlation between total Mφ and number of CD206+ Mφ for subclinical cows.

An assessment of correlations between mean numbers of total macrophages and macrophages that colocalized with cytokines or markers associated with the resolution and repair phenotype demonstrated significant and positive correlations with mean numbers of IL-1Ra+ macrophages (r = 0.898, p = 0.001) (Fig 6A), mean numbers of TGF-β+ macrophages for subclinical cows (r = 0.802, p = 0.009) (Fig 6C), and mean numbers of CD206+ macrophages for subclinical cows (r = 0.723, p = 0.018) (Fig 6D). In addition, total macrophages were correlated similarly with mean numbers of total IL-10+ macrophages for both subclinical (r = 0.877, p = 0.001) and clinical cows (r = 0.837, p = 0.003) (Fig 6B). Correlations were made between macrophages expressing markers of resolution and repair phenotype (i.e., CD163, CD206, IL-1Ra, IL-10, TNF-α, and TGF-β) within each disease state (Table 4). Again, subclinical cows demonstrated the most significant number of correlations (9 additional correlations, for a total of 13 correlations), with most common associations between macrophages colocalized with IL-10, IL-1Ra and TGF-β (Table 4). Of note, two correlations between macrophages colocalized with CD163 and IL-1Ra (r = -0.803, p = 0.005) were negatively correlated with IL-10 (r = -0.707, p = 0.022) for subclinical cows and were the only significant negative correlations observed in the study. Control cows demonstrated a total of 2 correlations not associated with total macrophage number, while clinical cows demonstrated only 1 additional correlation and that was between macrophages colocalized with IL-12 and macrophages colocalized with TNF-α (r = 0.971, p = 0.001) (Table 4).

**Table 4:** Pair-wise Pearson correlations between cytokines colocalized within resolution and repair macrophage phenotypes.

Dependent variable	Independent variable	Pearson correlation (r)	P-value
Control cows			
Mφ IL-1Ra	Mφ CD206	0.756	0.030
Mφ IL-23	Mφ uNOS	0.872	0.023
Subclinical cows			
Mφ ALL	Mφ IL-1Ra	0.898	0.000
Mφ ALL	Mφ IL-10	0.877	0.001
Mφ ALL	Mφ TGF-β	0.802	0.009
Mφ ALL	Mφ CD206	0.723	0.018
Mφ CD163	Mφ IL1-Ra	-0.803	0.005
Mφ CD163	Mφ IL-10	-0.707	0.022
Mφ IL-10	Mφ IL1-β	0.817	0.007
Mφ IL-10	Mφ CD206	0.639	0.047
Mφ IL-1Ra	Mφ IL-10	0.910	0.000
Mφ IL-1Ra	Mφ IL1-β	0.891	0.001
Mφ IL-1Ra	Mφ TGF-β	0.715	0.030
Mφ TGF-β	Mφ CD206	0.675	0.005
Mφ TGF-β	Mφ IL1-β	0.857	0.007
Clinical cows			
Mφ ALL	Mφ IL-10	0.837	0.003
Mφ IL-12	Mφ TNF-α	0.971	0.001

Abbreviations: Mφ, macrophage.

## Discussion

Macrophages are key phagocytic cells in the host response against infection with *Mycobacterium avium subsp*. *paratuberculosis* (MAP). In addition to the gastrointestinal system (GI) being the largest reservoir of macrophages in the body, the small intestine is the main gateway for MAP to enter the host. The role of intestinal macrophages in MAP infection is to kill the intracellular pathogen and to regulate concomitant inflammatory responses, however, MAP has developed strategies to disrupt the function and responsiveness of the macrophage. (5, 6)

Understanding the phenotypes of macrophages present is key to determining if the host can control the infection. Host defense macrophages are highly microbicidal, and once activated, can secrete various cytokines, including IL-1, IL-6, and IL-23 [22]. Wound-healing and regulatory (resolution and repair) macrophages originate from Th2-mediated immune responses in the host, and are generated in response to cytokines such as IL-4, IL-13, and IL-10, respectively [22].

In the present study, our key objective was to determine if there were measurable differences in the phenotype(s) of macrophages present in the target tissues of cows naturally infected with MAP during different stages of infection. Overall, a clear distinction between stage of disease and macrophage phenotype was observed. Clinically affected cows demonstrated significantly fewer numbers of host defense macrophages in the mid-ileum and similar numbers of regulatory macrophages when compared to both subclinical and control cows. The presence of a dominant Th2 immune response in clinically affected cattle in regards to significantly lower numbers of host defense macrophages is in agreement with previous studies on macrophage polarization and immune responses to MAP in cattle [5, 8, 23–24]. In contrast, noninfected control cows and cows that were subclinically affected had very similar numbers of both host defense and regulatory macrophages present in their tissue. The correlations between cytokines colocalized with macrophages further corroborated these results and lean towards a greater understanding of the infectious process. Using correlations between macrophages colocalized with cytokines, there was a definitive host defense phenotype present for control cows. Interestingly, subclinical cows demonstrated equivalent numbers of both host defense and resolution and repair macrophage phenotypes and, overall, had the highest number of correlations, regardless of phenotypic category. Finally, using these correlations, clinical cows demonstrated the least reactivity when using cytokine colocalization to assess a macrophage phenotype but did have 2 positive correlations that aligned with regulatory type macrophages.

For control cows, the majority of correlations involved macrophages that were either IFN-γ+ or IL-1β+, but also included macrophages positive for uNOS, IL-12 and IL-23, results that are consistent with a host defense macrophage phenotype. Previous studies have demonstrated an increase in the expression of genes encoding for IFN-γ in tissues and cells collected from MAP infected cattle [25–26], and MAP infected deer [27], as well as in the expression of genes encoding for IL-1β [26,28–29] and secretion of IL-1β protein [29]. In contrast, a difference in the relative fold change of IL-1β was not observed when comparing between severe disease, minimal disease and control red deer, although IL-1α expression was significantly increased in deer with severe disease [27]. It is possible that the lack of a difference between disease states in the study could be due to the lapse in time post-infection, as samples collected from severe disease animals were collected 25 weeks later than for control and minimal disease animals, as well as species differences [27]. However, it seems likely that in the noninfected host, macrophages may be at rest but their phenotype may be aligned with a “ready” status that would allow rapid response to invading pathogens, conforming to a greater presence of host defense macrophages.

Subclinical cows demonstrated relatively equivalent numbers of both host defense and regulatory macrophage phenotypes in the ileal tissue, findings that were further substantiated with the observed correlations of cytokine colocalization on macrophages. For host defense macrophage phenotype correlations, the majority of correlations involved macrophages colocalized with either IL-12 or IL-23. Both IL-12 and IL-23 play important roles in the inflammatory response in the tissues, however, IL-12 mediates inflammation through Th1 cells and IFN-γ production, whereas IL-23 drives the production of IL-17 and IL-22 through Th17 cells [30–31]. Previous studies demonstrated enhanced miRNA expression of IL-23 receptor in tissue of cattle, as well as an overexpression of IL-23 in the ileum of sheep infected with MAP, respectively [32–33]. The IL-12 p40 subunit forms part of the heterodimer IL-23 [34], so the highly significant correlation between IL-12 and IL-23 (r = 0.880, p = 0.004; Table 3) noted in the tissue of subclinically affected cows was unsurprising (Table 3). For the resolution and repair macrophage phenotype, correlations were observed between macrophages colocalized with IL-1Ra, IL-10, TGF-β, and CD206. Interleukin-1Ra, IL-10 and TGF-β play essential roles in controlling IL-1β activity and limiting inflammation through regulatory roles. A review of 12 transcriptional studies elucidating the signature of macrophages exposed to bacterial pathogens yielded a profile of gene upregulation associated almost exclusively with M1-type macrophages. The only exception was an upregulation of IL-1Ra, a gene associated with M2 polarization [1]. The association of IL-1Ra and macrophages from subclinical cows would suggest that persistent stimulation of the intestinal immune system by enteric bacteria at very low levels may initiate a polarization from M1 to M2 macrophages to control infection and thwart progression to more advanced disease. The presence of both host defense and resolution and repair macrophages in the tissue of subclinical cows was further demonstrated by significant positive correlations between IL-10+ macrophages and IL-12, IL-23, and uNOS (r = 0.87, 0.88, and 0.78, respectively; S1 Dataset) The concurrent upregulation of a regulatory cytokine such as IL-10 with the aforementioned pro-inflammatory mediators is consistent with a bipolar macrophage presence, suggesting this may be critical for how the host in the early stages of infection can not only limit MAP replication within the macrophage but also any associated inflammation.

Of note, subclinical cows demonstrated the only two negative correlations in the present study, both of which involved CD163. A negative correlation was observed between CD163 and IL-1Ra (r = -0.803, p = 0.005) and between CD163 and IL-10 (r = -0.707, p = 0.022) (Table 4). These correlations are interesting considering that all of these markers are associated with the repair and resolution macrophage phenotype. CD163 is a scavenger receptor that is associated with M2-like macrophages and mediates anti-inflammatory and anti-microbial events during bacterial infections [35]. Although there was no significant difference in the mean numbers of CD163+ macrophages between control and subclinical cows in the current study, CD163 could potentially be used as an indicator of a shift from the mixed Th1/Th2 immune state seen in subclinical cows, to the Th2 dominant immune state seen in clinical cows. This becomes more realistic with the recognition that only 10–15% of infected animals develop clinical disease [36], suggesting that the lack of a dominant Th1 or Th2 immune response in subclinical cows may control the infection. This is further substantiated by the use of CD163 as a potential marker for pulmonary TB in humans, with increased levels of CD163 associated with higher rates of mortality in patients with active TB [35]. In the present study, clinically affected cows had greater numbers of CD163+ macrophages compared to control and subclinical cows, providing further evidence that excessive inflammation induces CD163 expression.

Clinically affected cows in the advanced stage of disease had no cytokine correlations noted within host defense macrophages and correlations between IL-12 and TNF-α within resolution and repair phenotype macrophages. Previous studies in MAP infected animals have demonstrated increases in TNF-α gene expression in tissue [26,28] and cells [5], as well as higher histopathology score in tissues [23]. Previous studies have also demonstrated an increase in the expression of IL-10 in tissues and cells collected from clinical cows, as well as in macrophages incubated with MAP [24,37–38]. In a red deer infection model, expression of IL-10 trended upward in severely diseased animals but was not significant [24]. In vivo effects of infection on IL-12 have not been well-studied for MAP, however, in vitro studies suggest a lack of association between macrophages and IL-12 [37,39]. The positive correlation observed herein between IL-12+ macrophages and TNF-α+ macrophages is consistent with host defense macrophages stimulating the production of tissue repair macrophages (Fig 1).

Of particular interest in the present study was the observation of increased numbers of CD163+ macrophages concomitant with decreased numbers of CD206+ macrophages in clinical cows. Both types of macrophages are associated with the M2-like phenotype, so the dichotomous expression was puzzling. CD163 is a scavenger receptor for the hemoglobin-haptoglobin complex, but also functions as an innate immune sensor for both gram-positive and gram-negative bacteria [40]. Additionally, the interaction of CD163 with particular ligands results in either a strong anti-inflammatory response, or the release of pro-inflammatory mediators [40]. In a previous study, the proportion of bovine monocytes that expressed CD163 was significantly decreased in cows classed as high IFN-γ-responders when compared to low IFN-γ-responding cows [6]. In addition, a higher histopathology score was observed for macrophages expressing CD163 in diffuse multibacillary lesions (late-stage disease) [23]. The results of these previous studies are consistent with the concomitant down-regulation of IFN-γ and up-regulation of CD163 observed in clinical cows in the current study. It is likely that the increase in macrophages colocalized with CD163 in clinical cows is associated with the induction of anti-inflammatory mediators, resulting in the maintenance of infection and typical pathology observed in clinical cows, i.e. extensive inflammation and corrugation of the intestine.

CD206 is a mannose receptor (MR) C-type lectin found on monocytes and macrophages and facilitates antigen presentation via MHC Class II and CD1b pathways [41]. The reduced expression of CD206 on intestinal macrophages in clinically affected cows was surprising given its alignment with M2-like macrophages. In support of our finding, a lower mean fluorescence intensity of CD206 was demonstrated on macrophages collected from human *Mycobacterium tuberculosis* (Mtb) granulomas as compared to controls [42]. CD206 is also associated with controlling macrophage phagocytosis. Uptake of both MAP and Mtb via this receptor has been demonstrated to interfere with macrophage phagosome-lysosome fusion by interaction of the MR with mannosylated lipoarabinomannan (ManLAM), a cell wall component of pathogenic mycobacteria [43–45]. However, it should be noted that reduced phagosome-lysosome fusion has also been observed in monocytes without the MR [43–44]. The decrease in numbers of macrophages colocalized with CD206 in clinical cows suggests that MAP may be adequately phagocytized by these macrophages, killing of the mycobacterium was thwarted along with antigen presentation, leading eventually to less activation of T cells helping to clear the infection.

## Conclusions

In summary, the current study utilized immunofluorescence to identify macrophage phenotypes present in the mid ileum of cattle naturally infected with MAP. Additionally, the current study was also able to ascertain differences in numbers of macrophage phenotypes between different disease states, as well as relationships between macrophage phenotypes and local cytokine production. Clinical cows demonstrated significantly higher numbers of CD163+ and significantly lower numbers of CD206+ macrophages, although both markers are associated with M2-Like macrophages. Overall, clinical cows demonstrated significantly lower total numbers of macrophages with a host defense phenotype when compared to both subclinical cows and uninfected control cows. Additionally, subclinincal cows demonstrated a balance of host defense and resolution and repair phenotype macrophages, compared to dominant host defense and resolution and repair macrophage phenotypes in control and clinical cows, respectively.

The results of the current study offer insight into macrophage phenotypes present in the bovine ileum during different stages of infection. Current evidence suggests that macrophages of clinically infected cattle are unable to clear MAP infection, however, the reasons why this is the case has only partly been determined. Based upon the results of the current study, it is possible that the promotion of macrophages polarized towards resolution and repair, which favors maintenance of infection rather than the destruction of the infecting pathogen, is the cause of this inability to clear MAP. Although some results of the current study are in contrast to previous studies of cytokine expression, we believe that this study provides a more specific representation of the predominant macrophage phenotypes present in the bovine ileum of naturally infected cattle during the different stages of MAP infection.

## Supporting information

S1 Dataset Phenotype data- means per animal. (XLSX)
